# Editorial: Community series in mental health promotion and protection, volume II

**DOI:** 10.3389/fpsyt.2023.1291375

**Published:** 2023-10-03

**Authors:** Naseem Akhtar Qureshi, Harshavardhan Sampath, Samrat Singh Bhandari

**Affiliations:** ^1^Senior Consultant Psychiatrist, Sanjeevni Hospital, Jasola, New Delhi, India; ^2^Department of Psychiatry, Sikkim Manipal Institute of Medical Sciences, Sikkim Manipal University, Sikkim, India

**Keywords:** mindfulness, workplace mental health promotion, youth mental health care, machine-learning and AI, mental health

If you cannot fly then run, if you cannot run then walk, if you cannot walk then crawl, But whatever you do you have to keep moving forward.

— Martin Luther King Jr.

On this momentous occasion of the release of the second volume of the Community Series in Mental Health Promotion and Protection, the editorial team wishes to draw inspiration from the immortal quote of the civil rights activist Martin Luther King Jr. Witnessing the overwhelming reception to the first volume, we were under pressure to replicate our success. We wish to thank all the peer reviewers who despite their commitments took their invaluable time to review the articles. The tireless and dynamic efforts of the Frontiers publication team in coordinating this complex process needs special endorsement.

The breadth and depth of areas covered in this Research Topic span diverse populations, countries, methods and methodologies, and domains. The mental health of young adults, women, LGBTQ2+, elderly, their caregivers, healthcare workers, and students are extensively explored in this Research Topic. The areas researched are not limited to mental disorders but transcend to occupational, social, and spiritual wellbeing. Such diversity is a testimony to the originality, ingenuity, and perceptive insight of mental health professionals and academic researchers who have contributed to this Research Topic.

Steering across the experiences of menopause can be a journey filled with feelings of stress, anxiety and depression. One potential solution to prepare and travel the transition may be mindfulness-based interventions (MBIs) which are techniques that empower individuals to better handle their emotional wellbeing (1). A systematic review and meta-analysis conducted by Liu H. et al. explored how effective MBIs are, in reducing these symptoms and effect on mindfulness among women going through menopause. Although the findings indicated decreases in stress levels, further research is necessary to confirm their impact on anxiety, depression and mindfulness. By embracing MBIs, women may discover a pathway toward resilience during menopausal phase.

In a groundbreaking study led by Nawaz et al., the impact of #PsychTwitter on global psychiatry is unveiled. Twitter, a platform often characterized by its brevity, has proven to be a rich source of mental health discussions. The study demonstrates that the #PsychTwitter movement has generated a staggering 492,565,230 impressions, emphasizing its role as an important platform for disseminating mental health knowledge globally. Notably, this research also highlights the prominent role played by psychiatrists, academic organizations, and advocacy groups in shaping mental health discourse.

Recognizing the significance of youth mental health promotion, Jenkins et al. started on an innovative journey. They studied “Agenda Gap”, an intervention that allows young people to be involved in advocating policies designed to promote wellbeing and mental health. The youth involved have had a profound impact on their own mental health by raising their voices and making positive changes. Giving young people a voice and a platform to advocate for policies that address issues like mental health stigma, access to resources, and support systems, not only improves their own wellness but also helps build a more cohesive and resilient community (2). Youth mental health finds special focus in this Research Topic. Depression, anxiety, and stress experienced by school students of Bangladesh awaiting their entry into college after appearing for their entrance examination are captured by Rabby et al.. Dissatisfaction with physical appearance is a growing concern, particularly among youth in non-Western societies (3). Body dissatisfaction among Chinese University students, explored by Hao et al., found that female students had greater levels of dissatisfaction and it correlated significantly with depression, sleep quality, and physical activity.

An Iranian study on the social wellbeing of its citizens by Mahmoodi et al. reported a significant but weak positive correlation of mental health literacy (MHL) and Subjective Well Being (SWB) scores. Use of mental health services in the past or having someone familiar having mental illness was associated with poor SWB. While improved MHL can have positive effects on mental health outcomes, it is not a guaranteed predictor of high SWB. Even with good MHL, individuals who suffer from severe mental illness like schizophrenia may face enacted or perceived stigma, which may impair the SWB. An improved MHL may help individuals recognize the need for mental health support, but access to quality mental health services and resources can be limited in many countries (4). Other factors like lack of access to appropriate care, poor social support and economic hardship can contribute to poor SWB despite adequate MHL. The role of education and awareness about both mental illnesses and physical illnesses (5) has an important role to play in adherence and better prognosis.

The COVID-19 pandemic has cast a spotlight on mental health like never before. Multiple studies have examined its multifaceted impact, from the relationship between exposure and psychological distress during lockdowns (Liu Y. et al.) to the disparities in fear and anxiety experienced by different genders (Alibudbud). Chen et al. delved into the complex interplay between family context and psychological distress during the pandemic, highlighting the dual role families play as both a source of support and stress. Meanwhile, Ding et al. explored how the pandemic exacerbated insomnia symptoms in older adults and their caregivers. These studies emphasize the need for comprehensive mental health interventions including early diagnosis treatment and prevention in the face of global crises in future. The mental health professionals can promote mental wellbeing through different strategies during and after such crises (6).

Workplace mental health explored by Cabrera-Aguilar et al., among nurses in Peru reported that workplace engagement was mediated by self-efficacy, which had a positive impact on resilience and stress levels. These revelations have important ramifications for nursing management in healthcare institutions. It is essential that strategies are developed to support nurses' self-efficacy and resilience to promote a resilient and engaged nursing workforce. Harnessing the power of artificial intelligence, Kim et al., have used machine-learning models to predict depression in young Korean employees. The significance of the study lies in its potential to improve early diagnosis and prevention of workplace depression by providing specialized therapies and support networks.

A community-based survey in Ethiopia by Abdeta et al., on common mental disorders reported a prevalence of 21.31%, which was significantly associated with female gender, elderly, being single, widowed, and unemployed. Data from the Understanding Society: UK Household Longitudinal Study was used by Kang to conclude that psychological distress in angina sufferers was not limited to depression and anxiety but also independently affected social functioning, confidence levels, and ability to enjoy life. Using data on intimate partner violence, depression, and suicide in women across 151 countries, a population ecological study by Rajkumar found an association between cultural collectivism and intimate partner violence, which was significantly influenced by national income and women's educational level.

Mental health disorders affects people of all backgrounds and necessitates a shift toward proactive prevention and promotion. This involves fostering awareness, debunking stereotypes, and early education to equip individuals with emotional tools. Prevention also involves addressing risk factors such as adverse experiences and social disparities through policies and community support. Promotion goes beyond absence of disorders, empowering individuals to cultivate positive wellbeing through exercise, diet, mindfulness, and creative outlets. Workplaces should prioritize psychological safety and work-life balance, while society must collectively fund mental health services, enact policy changes, and destigmatize mental health concerns through advocacy and personal stories.

In Summary as William Shakespeare said “All's Well that Ends Well” and accordingly with the help of Chief Executive Officer and Frontiers publication department, we successfully completed the Research Topic on community series in *Mental health promotion and protection: volume II* which is ready for publication. For the knowledge of health researchers, mental health Professionals and Academicians, our editor team and frontiers have also launched Research Topic—*Developmental trajectories of early life trauma-volume III* which is getting good response from the research contributors at global level.

## Author contributions

NQ: Conceptualization, Writing—review and editing. HS: Conceptualization, Writing—original draft. SB: Conceptualization, Writing—original draft, Writing—review and editing.
